# Hemorrhagic Cystitis in Patients Who Underwent Allogeneic Stem Cell Transplantation (2012–2025): A Single‐Center Experience

**DOI:** 10.1155/ah/4650693

**Published:** 2026-09-17

**Authors:** Khanh Ba Nguyen, Binh Thi Thanh Vo, Nhung Thi Nguyen, Thanh Ha Nguyen

**Affiliations:** ^1^ Department of Hematology, Hanoi Medical University, Hanoi 100000, Vietnam, hmu.edu.vn; ^2^ Stem Cell Bank, National Institute of Hematology and Blood Transfusion, Hanoi 100000, Vietnam; ^3^ Stem Cell Transplantation Department, National Institute of Hematology and Blood Transfusion, Hanoi 100000, Vietnam

**Keywords:** allogeneic hematopoietic stem cell transplantation, BK virus, hemorrhagic cystitis, posttransplant cyclophosphamide, prognosis

## Abstract

**Introduction:**

Hemorrhagic cystitis (HC) is a common complication of allogeneic hematopoietic stem cell transplantation (HSCT), especially in cases with haploidentical donors. Our study aimed to determine the incidence and analyze the risk factors associated with HC in patients undergoing allogeneic HSCT.

**Subjects and Methods:**

This retrospective, cross‐sectional study analyzed data from 343 patients who underwent allogeneic HSCT between January 2012 and June 2025 at our institution. The research variables included clinical characteristics, conditioning regimens, BK virus (BKV) status, and transplant outcomes.

**Results:**

The overall incidence of HC was 18.4% (63 out of 343 patients), with severe cases (Grades 3‐4) comprising 9.4%. The median onset was 33 days posttransplantation, with a median duration of 40 days. BKV was detected in the urine of 19.9% of the patients, among whom 15.2% exhibited high viral loads. Multivariate Cox regression analysis identified three independent risk factors: malignant disease (hazard ratio [HR] = 4.212; *p* = 0.046), posttransplant severe infection (HR = 1.793; *p* = 0.033), and the use of posttransplant cyclophosphamide (PTcy) (HR = 3.404; *p* < 0.001). Regarding prognosis, the presence of HC did not significantly affect the 5‐year overall survival (OS) probability (67.1% vs. 65.7% in the non‐HC group, *p* = 0.921). However, patients with Grades 3‐4 HC exhibited a significantly lower 5‐year OS than those with Grades 1‐2 (49.6% versus 86.6%, *p* = 0.007).

**Conclusion:**

HC is a prevalent complication of allogeneic HSCT, often presenting late and closely associated with malignancies, severe infections, and PTcy protocols. The severity of HC is a crucial determinant of survival outcomes, necessitating proactive preventive strategies and timely interventions to enhance the patient’s prognosis.

## 1. Introduction

Hemorrhagic cystitis (HC) is a significant and potentially life‐threatening complication following allogeneic hematopoietic stem cell transplantation (HSCT) [1–3]. It is defined as an inflammatory condition of the bladder resulting from infectious or noninfectious etiologies that lead to bleeding of the bladder mucosa [4, 5]. The clinical manifestations of HC are diverse, ranging from mild, nonvisible microscopic hematuria to severe macrohematuria with clot formation, urinary tract obstruction, and subsequent renal failure [4–6]. Beyond the immediate physiological impact, HC significantly increases patient morbidity, prolongs the length of hospital stays, increases the requirement for transfusion support, and contributes to substantial healthcare costs [3–5, 7]. In the most severe cases, HC can be a direct or contributing cause of patient mortality [2, 4, 5].

The reported incidence of HC in the allogeneic setting varies widely across the literature, ranging from 7% to 54% in adult recipients and 8% to 25% in pediatric populations [5]. This broad range is often attributed to differences in definitions, conditioning intensities, donor types, and prophylactic strategies across various centers [1, 4]. Recent advances in transplant platforms, particularly the widespread adoption of haploidentical HSCT utilizing posttransplant cyclophosphamide (PTcy), have been associated with a notable increase in the frequency of HC [3, 8]. While PTcy has effectively reduced the risk of graft‐versus‐host disease (GVHD) in mismatched settings, it may contribute to HC through direct mucosal injury and by delaying the reconstitution of virus‐specific T‐cells, thereby facilitating uncontrolled BK virus (BKV) replication [3].

Despite the implementation of prophylactic measures such as hyperhydration, forced diuresis, and the use of mesna, HC remains a prevalent clinical challenge for which no universal gold‐standard management exists [2]. Most available evidence regarding therapeutic interventions, such as cidofovir, hyperbaric oxygen therapy, or cellular immunotherapy, is derived from small retrospective cohorts or case series, leading to variable efficacy results [3, 5]. Furthermore, the risk factors and clinical outcomes of HC can vary significantly depending on the specific protocols and patient demographics of a given institution [9].

Therefore, there is a clear need for center‐specific data to better characterize the burden of this complication and optimize local management protocols [4]. This study aimed to describe the incidence, severity, and risk factors of HC in 343 patients who underwent allogeneic stem cell transplantation at our single center. By identifying the clinical characteristics and outcomes of HC in our specific setting, we aim to contribute to the growing body of knowledge needed to establish more effective, evidence‐based guidelines for this debilitating condition.

## 2. Subjects and Methods

### 2.1. Study Design and Setting

We conducted a retrospective, cross‐sectional study to evaluate the incidence, risk factors, and clinical outcomes of HC in patients who underwent allogeneic HSCT. The study was conducted at the Department of Stem Cell Transplantation, National Institute of Hematology and Blood Transfusion (NIHBT). The study period spanned from January 2012 to June 2025, with retrospective data collection and analysis completed by September 2025.

### 2.2. Patient Selection and Eligibility

The study cohort comprised 343 consecutive patients who underwent allogeneic HSCT during the study period. The eligibility criteria included patients who underwent their first allogeneic HSCT and possessed comprehensive medical records from the initiation of conditioning through the posttransplant follow‐up period. The exclusion criteria encompassed patients with preexisting HC prior to the commencement of the conditioning regimen, those who underwent a second HSCT, and individuals in whom HC was clearly attributable to causes unrelated to the transplant procedure, such as bacterial urinary tract infections or drug‐induced toxicity from nontransplant protocols.

### 2.3. Transplantation and Prophylaxis Protocols

Conditioning regimens were categorized as myeloablative conditioning (MAC) or reduced‐intensity conditioning (RIC) according to institutional standards derived from common protocols in the world [10–13]. MAC regimens typically utilize intravenous busulfan and cyclophosphamide, whereas RIC regimens focus on fludarabine and reduced‐dose cyclophosphamide.

For GVHD prophylaxis, patients received various combinations of cyclosporine A, tacrolimus, methotrexate, and mycophenolate mofetil. A specific subset of patients, primarily in the haploidentical setting, received high‐dose PTcy on Days +3 and +4 posttransplantation. In cases of renal impairment, the cyclophosphamide dose was adjusted based on the estimated creatinine clearance (CrCl), specifically with a 25% reduction for patients with CrCl < 10 mL/min. To prevent acrolein‐induced urothelial damage, mesna was administered via continuous infusion at a total dose of twice the adjusted daily cyclophosphamide dose, along with aggressive hyperhydration and forced alkaline diuresis to maintain a target urine output of > 200–250 mL/h.

### 2.4. Definitions and Grading of HC

HC is characterized by irritative urinary symptoms, such as dysuria, urgency, or abdominal pain, in conjunction with hematuria. Cases are classified as early‐onset if they occur within 72 h of conditioning and late‐onset if they manifest after 72 h postinfusion. The severity of HC is graded according to the National Cancer Institute (NCI) and European Conference on Infections in Leukemia (ECIL) criteria: Grade 1 involves microscopic hematuria, Grade 2 involves macroscopic hematuria, Grade 3 involves macroscopic hematuria with visible clots, and Grade 4 involves macroscopic hematuria with urinary tract obstruction or renal impairment [1, 5]. Consistent with established guidelines, severe HC was defined as Grade 3 or higher [5].

Urinalysis was performed from the onset of HC symptoms to 100 days following transplantation. In patients with symptoms, urinary BKV DNA was quantified using a real‐time polymerase chain reaction (qPCR) assay. Viral DNA was extracted from the urine samples using an automated nucleic acid extraction system. Amplification and quantification were performed on a Roche Cobas system using specific primers and TaqMan probes targeting the conserved VP1 or Large T‐Antigen (LTA) gene regions. High‐level BK viruria was characterized by a viral load exceeding 10^7^ copies/mL [5]. BKV DNA was quantified once at the time of symptom appearance and only redone if the initial result was negative, but the symptom persisted without proper explanation. Cytomegalovirus (CMV) reactivation was monitored by qPCR twice a week after stem cell transfusion, using EDTA‐coagulated blood. Engraftment was defined by the standard criteria of an absolute neutrophil count (ANC) and platelet count unsupported by transfusion [14].

### 2.5. Statistical Analysis

Data for this study were obtained from both physical medical records and the hospital’s existing electronic databases. The analysis was conducted using SPSS Version 20.0. Descriptive statistics were used to delineate the demographic and clinical characteristics of the study population. For the survival analyses, the Kaplan–Meier method was utilized for two distinct endpoints: 5‐year overall survival (OS), with death from any cause defined as the event, and the cumulative incidence of HC, with the onset of HC symptoms defined as the event. Differences in survival and incidence curves between groups were assessed using the log‐rank test. To identify potential risk factors associated with HC development, univariate analysis was performed using univariate Cox proportional hazards regression models for both categorical and continuous variables. Variables that demonstrated statistical significance in the univariate analysis (*p* < 0.2), along with clinically relevant baseline characteristics, were subsequently included in a multivariate Cox proportional hazards regression model to ascertain the independent predictors of HC. Patients who died or were lost to follow‐up prior to Day +30 were excluded from survival analysis. Statistical significance for all analyses was defined as *p* < 0.05.

### 2.6. Ethical Considerations

The study was approved by the Institutional Review Board and Ethics Committee of the NIHBT under approval number 45/HHTM. All patient‐identifying information was anonymized and encrypted. The data were used solely for scientific research purposes, ensuring that the study posed no risk or adverse impact on the participants. The research team rigorously adhered to the principles of confidentiality, data protection, and integrity throughout data collection and analysis.

## 3. Results

The average age of the patients was 28.3 years, with ages spanning from 3 to 60 years, and children constituted 23.9% of the total participants (Table 1). The majority of the group (74.3%) had malignant conditions, primarily acute leukemia, while 25.7% had nonmalignant disorders, such as aplastic anemia (Table 1). The most frequently used stem cell source was 10/10‐matched peripheral blood, accounting for 63%, followed by haploidentical peripheral blood (25.7%) and cord blood (11.3%). Conditioning regimens often included busulfan in 79.6% of cases and cyclophosphamide in 84.8% of cases (Table 2).

**TABLE 1:** General characteristics of the study population (*n* = 343).

	*n*	%
Age		
≥ 18 years old	261	76.1
< 18 years old	82	23.9
Gender		
Male	200	58.3
Female	143	41.7
Malignant diseases	255	74.3
‐ Acute leukemia	192	56.0
‐ Myelodysplastic syndrome	23	6.7
‐ Lymphoma	15	4.4
‐ Chronic myeloid leukemia	13	3.8
‐ Chronic myelomonocytic leukemia	9	2.6
‐ Myeloproliferative neoplasms	3	0.9
Nonmalignant diseases	88	25.7
‐ Aplastic anemia	61	17.8
‐ Thalassemia	19	5.6
‐ Paroxysmal nocturnal hemoglobinuria	8	2.3

**TABLE 2:** Characteristics of the stem cell transplantation process (*n* = 343).

	*n*	%
Stem cell source		
10/10 matched peripheral blood	216	63.0
Haploidentical peripheral blood	88	25.7
Cord blood	39	11.3
Conditioning agents		
Busulfan	273	79.6
Cyclophosphamide	291	84.8
Fludarabine	130	37.9
ATG	95	27.7
Etoposide	40	11.7
GVHD prophylaxis		
Cyclosporine	252	73.5
Tacrolimus	91	26.5
PTcy	86	25.1

*Note:* PTcy, posttransplant cyclophosphamide.

Abbreviations: ATG, antithymocyte globulin; GVHD, graft‐versus‐host disease.

Based on the Kaplan–Meier survival analysis, the cumulative incidence rate of HC increased over time, reaching a maximum estimated rate of 18.4% by Day 83 posttransplantation and remained constant until the end of the 100‐day follow‐up period. Among those affected, Grade 3 was the most common severity level, representing 8.2% of the entire group, followed by Grade 2 at 4.7% (Table 3). BKV was found in the urine of 19.9% of all patients, with higher viral loads observed in 15.2% of them (Table 3). The median time for symptoms to onset was 33 days after transplantation, and symptoms lasted for a median of 40 days. HC was notably more frequent in patients who had haploidentical transplants (39.8%) and those with malignant conditions (22.4%) (Table 3).

**TABLE 3:** Characteristics of hemorrhagic cystitis (*n* = 343).

	*n*	%	Kaplan–Meier cumulative incidence (95% CI)
No symptoms of cystitis	268	78.1	
Symptoms of cystitis	75	21.9	
Hemorrhagic cystitis	63	18.4	18.4% (14.3%–22.5%)
‐ Grade 1	15	4.3	
‐ Grade 2	16	4.7	
‐ Grade 3	28	8.2	
‐ Grade 4	4	1.2	
Nonhemorrhagic cystitis	12	3.5	
Testing of pathogen			
Positive urine bacterial culture	9	2.7	
Positive urine BK virus	68	19.9	
‐ Low viral load	16	4.7	
‐ High viral load	52	15.2	
Clinical course of HC			Median (Min–Max)
Time to onset from transplant (days)	—	—	33 (5–83)
Duration of symptoms (days)	—	—	40 (5–237)
Mean time to remaining event‐free (days)	—	—	87.59 (95% CI: 84.71–90.48)

Abbreviations: CI, confident interval; HC, hemorrhagic cystitis.

This study identified several factors associated with the development through univariate and multivariate analyses. Univariate factors, such as malignant disease, acute GVHD, severe infection, the use of busulfan, antithymocyte globulin (ATG), and PTcy, as well as CMV reactivation, were shown to have an impact. Multivariate analysis revealed that only three factors maintained independent statistical significance: malignant disease [hazard ratio (HR) = 4.212], posttransplant severe infection (HR = 1.793), and PTcy use (HR = 3.404) (Table 4).

**TABLE 4:** Univariate and multivariate Cox regression analyses of risk factors associated with hemorrhagic cystitis (*N* = 343).

Prognostic factor	*n*	%	Univariate analysis		Multivariate analysis	
			HR (95% CI)	*p* value	HR (95% CI)	*p* value
Gender (male)	200	58.3	0.955 (0.58–1.572)	0.856	0.858 (0.519–1.420)	0.551
Age (< 18)	82	23.9	0.823 (0.447–1.514)	0.531	0.971 (0.472‐1.996	0.936
Malignant disease	255	74.3	3.503 (1.510–8.126)	0.003	4.212 (1.024–17.321)	0.046
ATG conditioning	95	27.7	0.533 (0.278–1.022)	0.058	2.740 (0.950–7.906)	0.062
Busulfan conditioning	273	79.6	3.123 (1.253–7.787)	0.015	1.334 (0.317–5.615)	0.695
PTcy	86	25.1	4.505 (2.737–7.412)	< 0.001	3.404 (1.953–5.931)	< 0.001
CMV reactivation	255	74.3	5.630 (2.045–15.501)	0.001	2.440 (0.833–7.151)	0.104
Acute GVHD	95	27.7	1.950 (1.184–3.212)	0.009	1.415 (0.847–2.364)	0.185
Severe infection	147	42.9	2.707 (1.612–4.545)	< 0.001	1.793 (1.049–1.049)	0.033

*Note:* CMV, cytomegalovirus; PTcy: posttransplant cyclophosphamide.

Abbreviations: ATG, antithymocyte globulin; CI, confidence interval; GVHD, graft‐versus‐host disease; HR, hazard ratio.

**FIGURE 1:**
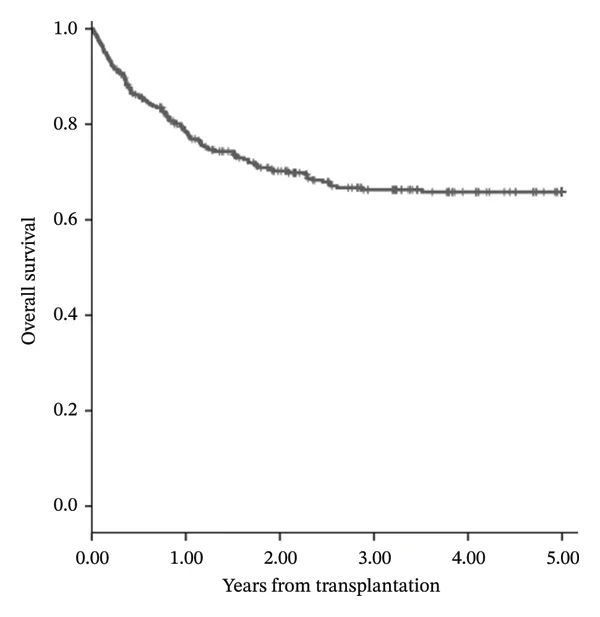
Five‐year overall survival after stem cell transplantation.

**FIGURE 2:**
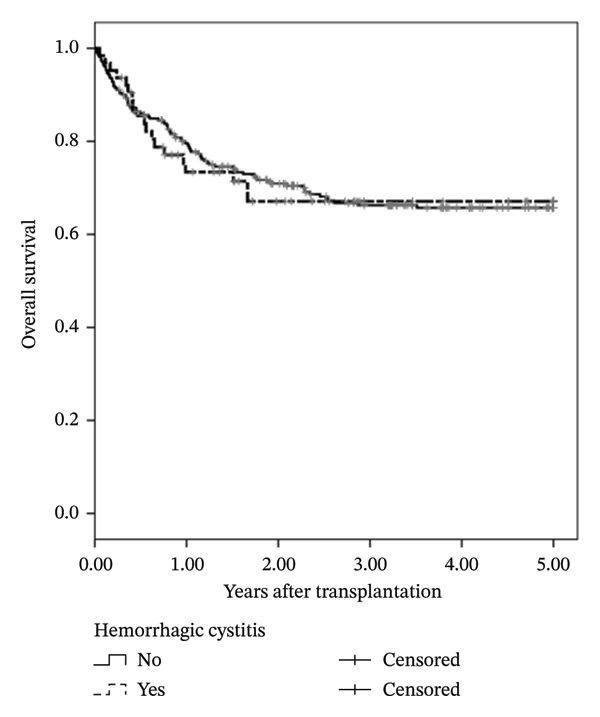
Overall survival according to the occurrence of hemorrhagic cystitis.

**FIGURE 3:**
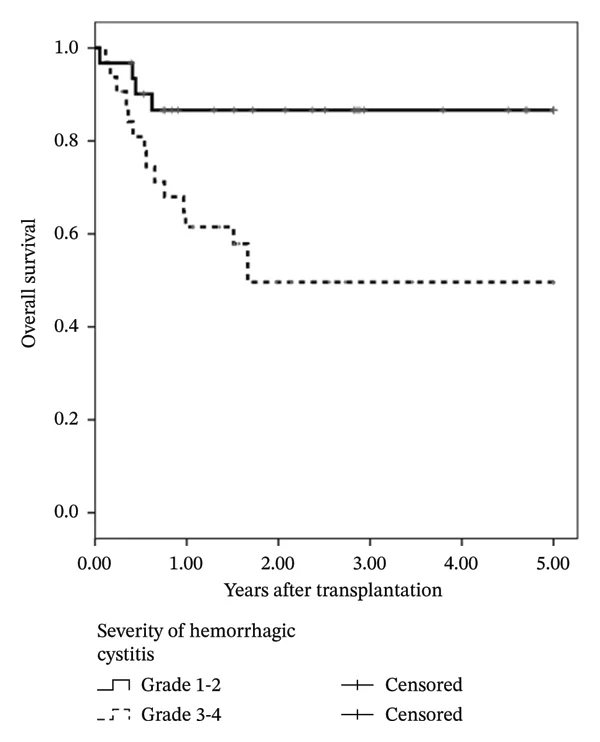
Cumulative probability of survival in patients with severe and nonsevere hemorrhagic cystitis.

The 5‐year OS probability for the entire cohort was 65.8% (Figure 1). The presence of HC did not significantly influence the overall 5‐year survival probability, with 67.1% for the HC group compared with 65.7% for the non‐HC group (*p* = 0.921) (Figure 2). However, HC severity is a critical prognostic factor. Patients with Grades 3‐4 HC had a significantly lower 5‐year OS of 49.6%, compared with 86.6% for those with Grades 1‐2 HC (*p* = 0.007) (Figure 3).

## 4. Discussion

In the present study, the overall cumulative incidence rate of HC, estimated using the Kaplan–Meier method, was 18.4%. This finding is notably in line with other studies, which have reported a range of 7% to 54% in adults and 8% to 25% in children [3, 5]. Specifically, our findings align with the 15.7% and 19% figures reported by Dosin et al. and Laskin et al. in studies involving pediatric populations [15, 16].

In the context of haploidentical transplantation, we noted a significantly higher occurrence rate of 39.8%. This finding aligns with the “PTcy era” studies, where Mukherjee et al. documented a 40% incidence and Ruggeri et al. reported 62% [7, 17]. The reason for this consistency is the combination of rigorous conditioning and the administration of PTcy. Although PTcy is very effective in preventing GVHD, it serves as an additional impact on the bladder mucosa, which has already been affected by the initial conditioning regimen [3, 18].

In this study, the median onset of HC was found to be 33 days, which is consistent with the findings of Arai et al. (35 days) and Zhang et al. (37 days) and falls within the 2‐ to 8‐week period identified as late‐onset HC in other research [1, 3, 5, 6, 19].

The biological mechanism underlying this timing is linked to immune reconstitution. Late‐onset HC typically occurs during the periengraftment period [5]. As donor immune cells begin to recover, they infiltrate the bladder wall, which has been denuded by viral replication (primarily by the BKV) and chemical injury [3, 5]. This “immune reconstitution inflammatory syndrome” (IRIS) effectively transforms asymptomatic viral shedding into clinical HC [5].

Regarding duration, our study reported a median of 40 days, which is significantly longer than the 14–20 days reported by Dosin et al. and the GITMO network [4, 16]. This discrepancy likely reflects the severity of the patient cohort, where Grades 3 and 4 cases were prevalent (9.4% combined). Severe cases are known to have a protracted course owing to the time required for urothelial regeneration in a profoundly immunosuppressed host [1, 5].

Our research identified BKV in 19.9% of the total population, with a high BKV load in 15.2%. This supports the ECIL diagnostic triad, which identifies a BKV load of > 7 Log_10_ copies/mL as the defining feature of BKV‐HC [3, 5].

A point of disagreement in the literature concerns the role of CMV. In our study, CMV reactivation was a significant risk factor in the univariate analysis but did not maintain independent significance in the multivariate model. In contrast, Zhang et al. found CMV infection to be the only independent variable significantly associated with HC (HR = 5.57) [19].

The mechanism underlying this disagreement may be explained by viral synergy. As noted by Ruggeri et al. and Jandial et al., CMV reactivation often precedes HC onset and serves as a surrogate marker for profound T‐cell lymphopenia [3, 7]. CMV does not necessarily cause cystitis directly but rather suppresses the host immune system, allowing BKV to replicate unchecked [9]. The variance between authors likely depends on the specific immunosuppression protocols and the aggressiveness of GVHD management.

Multivariate analysis identified malignant disease and PTcy use as the primary independent risk factors. This finding agrees with that of Arai et al., who noted that progressive disease status is a risk factor, potentially due to the higher total dose of chemotherapy, these patients received prior to transplant [1]. Our emphasis on PTcy is echoed by Mukherjee et al. and Jandial et al. [3, 17]. This dual mechanism involves acrolein toxicity and delayed T‐cell recovery [3, 18]. Interestingly, Jaiswal et al. reported a much lower HC incidence (5.4%) despite using PTcy, which they attributed to extended continuous mesna infusion [8]. This suggests that the method of drug delivery may mitigate the risks identified in our study.

We used mesna at twice the cyclophosphamide dose, combined with continuous infusion. This aggressive approach aligns with the findings of Mac et al. (2021), who also used high‐dose mesna (320% of PTcy dose) and reported a lower incidence of severe HC (Grade 3: 11.5%) than that of standard protocols [20].

The biological mechanism relies on the neutralization of acrolein. Acrolein is a toxic byproduct of cyclophosphamide that causes swelling and ulceration of the bladder urothelium [18]. Arango and Cardona confirmed that continuous infusion of mesna is superior to bolus dosing because acrolein is continuously produced as cyclophosphamide is metabolized; therefore, protection must be constant to prevent mucosal injury [3, 18].

Our data indicate that HC alone does not impact the 5‐year OS (67.1% vs. 65.7%). This is a point of wide agreement across the sources, including Ruggeri et al. and Dosin et al. [7, 16]. However, a critical distinction emerged in the severity analysis. We found that Grade 3‐4 HC patients had a significantly lower OS (49.6%) than Grade 1‐2 patients (86.6%). This mirrors the results of Arai et al., who reported a poor OS of only 16.7% in severe cases [1].

The mechanism of mortality in severe HC is rarely due to hemorrhage itself. Instead, severe HC is a marker of severe acute GVHD and opportunistic infections [1]. Patients with Grade 3‐4 HC often require surgical intervention, which further complicates their clinical status, leading to a downward spiral of renal impairment and sepsis [1, 4, 21].

The principal limitation of this study is its retrospective design, which spans an extensive period from 2012 to 2025. Due to the considerable timeframe covered, certain clinical information and laboratory results from earlier years were not fully preserved. As a result, this incomplete historical data have constrained the ability to conduct in‐depth specialized analyses concerning the correlations between the research variables and their associated factors. Secondly, owing to the retrospective design of the study and the reactive nature of our institutional BKV testing protocol, follow‐up quantitative PCR testing was not performed at uniform intervals across all patients, and testing was generally discontinued upon clinical resolution. Therefore, a precise calculation of the total duration of BKV viruria could not be established without introducing significant bias. The potential correlation between the kinetic duration of viral shedding and clinical HC severity remains to be elucidated in future prospective trials with standardized longitudinal monitoring protocols.

## 5. Conclusion

This research supports the common belief that HC following transplantation is a frequent complication, with its impact on survival depending on its severity. The study identified PTcy treatment, malignant conditions, and severe infections as independent risk factors that notably increased the likelihood of this complication. Although CMV reactivation and the administration of busulfan and/or ATG did not independently affect the incidence of this complication in our findings, they are still considered potential risk factors in many studies, highlighting the need for further investigation.

## Funding

This research received no funding.

## Disclosure

All authors have read and approved the final version of the manuscript. Dr. Khanh Ba Nguyen had full access to all of the data in this study and takes complete responsibility for the integrity of the data and the accuracy of the data analysis.

## Conflicts of Interest

The authors declare no conflicts of interest.

## Data Availability

The data that support the findings of this study are available from the corresponding author upon reasonable request.
